# Editorial: Interferon and its antiviral effect in response to HBV infection

**DOI:** 10.3389/fimmu.2023.1135649

**Published:** 2023-02-02

**Authors:** Hongxiao Song, Yongye Huang, Chunfeng Li, Quan Liu, Guangyun Tan

**Affiliations:** ^1^ Department of Hepatology, Infectious Diseases and Pathogen Biology Center, Institute of Translational Medicine, The First Hospital of Jilin University, Changchun, Jilin, China; ^2^ College of Life and Health Sciences, Northeastern University, Shenyang, China; ^3^ Institute for Immunity, Transplantation and Infection, Stanford University, Stanford, CA, United States; ^4^ Department of Infectious Diseases, Infectious Diseases and Pathogen Biology Center, Institute of Translational Medicine, The First Hospital of Jilin University, Changchun, China

**Keywords:** interferon, HBV - hepatitis B virus, ISGs, ISGs (IFN-stimulated genes), CccDNA, HCC (hepatic cellular carcinoma)

The disease caused by hepatitis B virus (HBV) infection is a universal and important health issue that troubles human beings. According to WHO (World Health Organization) estimates, about 2 billion people around the world have been infected with HBV, and about 20% of these infections become chronic, which can induce hepatocellular carcinoma (HCC). About 887,000 people worldwide die of HBV infection-related diseases per year, of which 52% are related to liver cirrhosis and 38% to hepatocellular carcinoma. Although widespread vaccination against hepatitis B has reduced infection rates, there are still nearly 100 million people carrying HBV and the number of carriers is still growing. HBV is a hepatotropic DNA virus with a genome of about 3.2 kb. It forms a covalently closed circular transcription template cccDNA in the nucleus and encodes four genes: S, X, P, and C. The S gene encodes three S virus envelope proteins: Small S (surface antigen: HBsAg), Middle S, and Large S. The X gene encodes regulatory protein HBx, which is related to cccDNA transcription. The P gene encodes virus DNA polymerases. The C gene encodes the core protein (Core) that forms the core particle of the virus, and the pre-core protein (Pre-core), which is the precursor of the secreted e antigen (HBeAg) (1).

Interferon (IFN), especially type I IFN, is the first line of defense of the human immune response. After infection, the virus is recognized by sensors inside and outside the cells, which activate the production of downstream interferon. After interferon production, it will recognize the interferon receptors on the surface of the cell membrane, leading to the activation of the JAK-STAT signaling pathway, and ultimately leading to the expression of hundreds of interferon-induced genes, including mRNAs and non-coding RNAs. These genes can directly or indirectly inhibit infection and the replication of viruses (2). However, unlike other viruses, HBV is relatively inefficient at inducing innate cytokines such as type I IFN in response to viral infections, which may be related to the fact that limited HBV-related sensors exist in the hepatocytes, and the expressed protein of HBV can inhibit the interferon signaling pathway (3). However, it has been reported that type III interferon can be induced after HBV infection, thereby inhibiting the replication of HBV (4). Interferon and its inducible genes can indeed inhibit the replication of HBV (5); this is also the reason why interferon is one of the two important drugs used for treating HBV- related diseases. Most members of many well-known gene families (such as the APOBEC and TRIM families) are interferon- inducible genes, which can significantly inhibit HBV infection and replication (6). However, not all interferon inducible genes can resist the virus, such as IFIT3, a classical ISG. After HBV infection, NF-κB can be activated through HBx, thereby promoting the expression of IFIT3, while IFIT3 can promote HBV replication (7). Some other HBx-interacted ISGs might also benefit the HBV lifecycle (6). In addition, IFN-α stimulation was reported to interfere with multiple intracellular signaling pathways, which facilitated autophagy initiation, and blocked autophagic degradation, thereby resulting in promoting HBV replication (8). Thus, the question is: confrontation between Interferon and HBV: friend or foe? (**Figure 1**).

**Figure 1:**
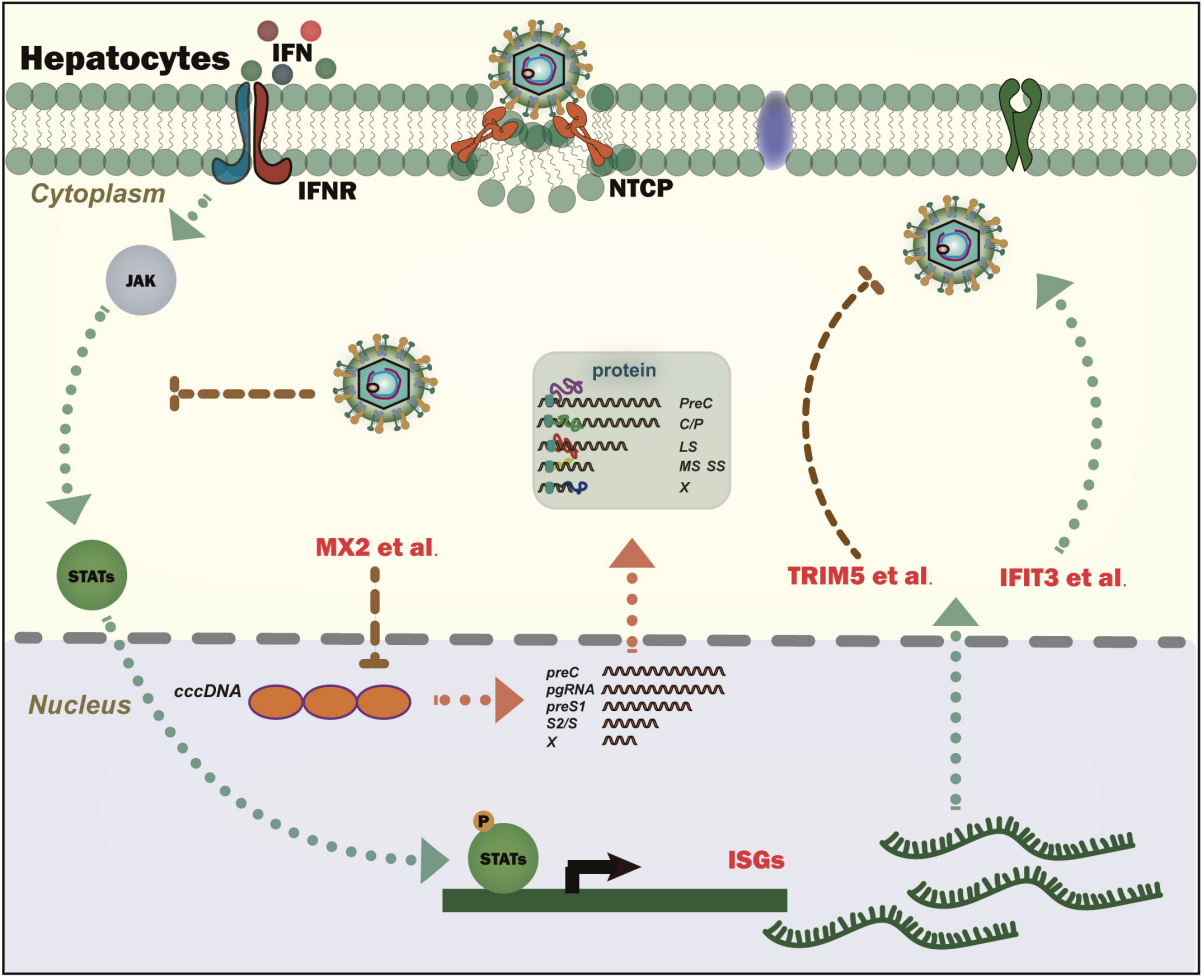
Model depicting the dual role of interferon in regulating the replication of the hepatitis B virus. Interferon stimulation results in the production of ISGs. Most ISGs play a positive role in regulating HBV replication; however, contrarily, some of them also promote HBV replication. ISGs (MX2 et al.) could reduce the HBV template cccDNA. Interferon signaling pathways are also inhibited by HBV proteins like HBx.

The key to the complete cure of chronic hepatitis B (CHB) is to clear the HBV transcription template located in the nucleus of the liver: covalent closed circular DNA (cccDNA). At present, there is a lack of therapeutic drugs directly targeting cccDNA (9). Interferon and nucleoside analogs, as mainstream CHB treatment drugs, have major defects such as drug resistance and serious side effects. The latest research shows that it only takes a few months to update the cccDNA library, which is much shorter than previous predictions (10). Therefore, blocking the complement of cccDNA in the nuclei of hepatocytes and reducing the existing cccDNA as soon as possible is an important means to achieve HBV clearance. Therefore, to clarify the interaction mechanisms between the host and HBV, developing effective small molecules and other therapeutic drugs, inhibiting or blocking HBV infection and replication, and finally eliminating cccDNA are the key basis for curing chronic hepatitis B.

It should be emphasized that interferon and its stimulated genes have the ability to reduce cccDNA levels in the nucleus of hepatocytes and, as previously mentioned, it remains unclear whether interferon is friend or foe. Thus, maintaining interferon’s “antiviral” essence while eliminating its “side effects” is our next mission.

## Author contributions

HS drafted the manuscript; GT revised the draft; YH and QL made substantial contributions to the work through in-depth discussion. All the authors proposed the Research Topic theme, made a direct and intellectual contribution to the work, and approved the final version for publication.
